# Lingguizhugan decoction improves non-alcoholic steatohepatitis partially by modulating gut microbiota and correlated metabolites

**DOI:** 10.3389/fcimb.2023.1066053

**Published:** 2023-01-26

**Authors:** Mingzhe Zhu, Xue Wang, Kai Wang, Zhiqiang Zhao, Yanqi Dang, Guang Ji, Fenghua Li, Wenjun Zhou

**Affiliations:** ^1^ Institute of Digestive Diseases, Shanghai University of Traditional Chinese Medicine, Shanghai, China; ^2^ School of Public Health, Shanghai University of Traditional Chinese Medicine, Shanghai, China; ^3^ Experiment Center for Science and Technology, Shanghai University of Traditional Chinese Medicine, Shanghai, China

**Keywords:** Lingguizhugan decoction, NASH, MCD diet, gut microbiota, metabolomics

## Abstract

**Background:**

Lingguizhugan decoction is a traditional Chinese medicine prescription that has been used to improve non-alcoholic fatty liver disease and its progressive form, non-alcoholic steatohepatitis (NASH). However, the anti-NASH effects and underlying mechanisms of Lingguizhugan decoction remain unclear.

**Methods:**

Male Sprague-Dawley rats were fed a methionine- and choline-deficient (MCD) diet to induce NASH, and then given Lingguizhugan decoction orally for four weeks. NASH indexes were evaluated by histopathological analysis and biochemical parameters including serum alanine aminotransferase (ALT), aspartate aminotransferase (AST), liver triglycerides (TG), *etc.* Fecal samples of rats were subjected to profile the changes of gut microbiota and metabolites using 16S rRNA sequencing and ultra-performance liquid chromatography coupled to tandem mass spectrometry (UPLC-MS). Bioinformatics was used to identify Lingguizhugan decoction reversed candidates, and Spearman’s correlation analysis was performed to uncover the relationship among gut microbiota, fecal metabolites, and NASH indexes.

**Results:**

Four-week Lingguizhugan decoction treatment ameliorated MCD diet-induced NASH features, as evidenced by improved hepatic steatosis and inflammation, as well as decreased serum AST and ALT levels. Besides, Lingguizhugan decoction partially restored the changes in gut microbial community composition in NASH rats. Meanwhile, the relative abundance of 26 genera was significantly changed in NASH rats, and 11 genera (such as *odoribacter, Ruminococcus_1*, *Ruminococcaceae_UCG-004, etc.*) were identified as significantly reversed by Lingguizhugan decoction. Additionally, a total of 99 metabolites were significantly altered in NASH rats, and 57 metabolites (such as TDCA, Glutamic acid, Isocaproic acid, *etc.*) enriched in different pathways were reversed by Lingguizhugan decoction. Furthermore, Spearman’s correlation analyses revealed that most of the 57 metabolites were significantly correlated with 11 genera and NASH indexes.

**Conclusion:**

Lingguizhugan decoction may exert protective effects on NASH partially by modulating gut microbiota and correlated metabolites.

## Introduction

Non-alcoholic steatohepatitis (NASH) is a severe form of non-alcoholic fatty liver disease (NAFLD), characterized by the presence of liver inflammation and hepatocyte injury (ballooning) due to fat accumulation (Fraile et al., 2021). NASH may progress into cirrhosis and hepatocellular cancer (HCC) and is presently a leading cause of liver transplantation, which has become a major health concern worldwide (Margini and Dufour, 2016; Raza et al., 2021). However, there are currently no approved effective medicines for NASH, so appropriate therapeutic approaches are urgently warranted.

Traditional Chinese Medicines (TCMs) have been proven to effectively treat hepatic diseases for centuries, attracting increasing attention for the treatment of NAFLD/NASH (Dai et al., 2021). In the past decades, TCMs have been reported to improve NAFLD/NASH through multiple mechanisms, including regulating lipid and glucose metabolism, improving liver inflammation, and protecting liver functions. For example, *Radix Polygoni Multiflori* and its main component emodin can attenuate NAFLD and hepatic steatosis (Wang et al., 2017; Yu et al., 2020). Berberine, an alkaloid component isolated from the traditional Chinese herbal *Coptidis Rhizoma*, is also considered to have therapeutic potential for NAFLD/NASH in both clinical investigations and animal studies (Zhou et al., 2017; Wah Kheong et al., 2017; Harrison et al., 2021). Gegenqinlian decoction abated NASH through anti-oxidative stress and anti-inflammatory response by inhibition of the toll-like receptor 4 (TLR4) signaling pathway (Zhang et al., 2020). Recently, growing evidence suggested that gut microbiota and related metabolites might play an important role in the therapeutic effects of TCMs on NAFLD/NASH. In particular, several TCMs could ameliorate NAFLD by regulating intestinal microbiota and its derived metabolites, such as short-chain fatty acids (Yu et al., 2020), branched-chain amino acids (Zhang et al., 2018), and bile acids (Li et al., 2021). Consequently, gut microbiota and correlated metabolites have emerged as novel targets for potential TCMs intervention in NAFLD.

Lingguizhugan decoction (LGZG) is an ancient Chinese herbal formula, which is composed of *Poriacocos* (*Schw.*) Wolf (Fuling in Chinese)*, Cinnamomum cassia* Presl (Guizhi in Chinese)*, Atractylodes macrocephala* Koidz. (Baizhu in Chinese)*, and Glycyrrhiza uralensis* Fisch. (Gancao in Chinese). In our recent randomized, double-blinded, placebo-controlled trial, low-dose LGZG effectively improved insulin resistance in overweight/obese NAFLD patients (Xu et al., 2020; Dai et al., 2022), which might be related to the regulation of DNA N6-methyladenine modification of protein phosphatase 1 regulatory subunit 3A (PPP1R3A) and autophagy-related 3 (ATG3) (Dai et al., 2022). Our previous study demonstrated that LGZG treatment significantly attenuated HFD-induced NAFLD probably through increasing serum thyroid hormone levels, improving fatty acid β-oxidation (via modulation of thyroid hormone receptor β1 and carnitine palmitoyltransferase-1A expression), and inhibiting the metabolism and transport (through modulation of sterol regulatory element-binding protein 1c, long-chain acyl-CoA synthetase, and Apolipoprotein B100 expressions) of fatty acids (Liu et al., 2013). Besides, LGZG has also been reported to improve oxidative stress, which is an independent risk factor that drives the progression of NAFLD to NASH called the “Two Hit Theory” (Zhang et al., 2015). However, the effect of LGZG on NASH and the underlying mechanism from the perspective of gut microbiota and related metabolites remained unclear.

In the present study, we used a rat model of methionine- and choline-deficient (MCD) diet-induced NASH to evaluate the effect of LGZG on NASH. Moreover, 16S rRNA sequencing and UPLC-MS technologies were used to profile the changes in gut microbiota and metabolites in fecal samples. The results might broaden our knowledge of the efficacy and underlying mechanisms of LGZG in treating NASH, and provide candidate microbiota and metabolites to improve NASH.

## Material and methods

### Preparation of Lingguizhugan decoction

Lingguizhugan decoction (powder batch number: Z201101) was provided by Professor Tong Zhang, School of Pharmacy, Shanghai University of Traditional Chinese Medicine. *Poriacocos* (*Schw.*) Wolf (batch number: Y2003002), *Cinnamomum cassia* Presl (batch number: 200608), *Atractylodes macrocephala* Koidz. (batch number: YP200601), and *Glycyrrhiza uralensis* Fisch. (batch number: YP200601) in a 2:1.5:1:1 ratio, added 12 times the amount of water, decoction 2 times, every 1.5 hours, collected the first water decoction, and set aside. Decoction filtered, combined two filtrates, the filtrate concentrated to a relative density of 1.07 ~ 1.09 (65 ± 5°C), spray-dried, and crushed into a fine powder for use (4.56 g crude medicine extracted 1 g extract powder, which was stored in a dry environment). All herbs were purchased from Jiangsu Sanhexing Chinese Medicine Research Co., Ltd. (Jiangsu, China). The fingerprint was used to control the quality of the LGZG (**Supplementary Figure 1**).

### Experimental animals

Male Sprague-Dawley rats (8-week-old, 300-350 g), were purchased from Shanghai Jihui Experimental Animal Technology Co., Ltd. (Shanghai, China), and maintained in the Laboratory Animal Center of Shanghai University of Traditional Chinese Medicine, which were kept in a constant temperature and humidity with a 12 h light/dark cycle. After one week of adaptive feeding, the rats were randomly divided into two groups. The Control group (n = 6) was fed a chow diet, and the model group was fed an MCD diet (Lot No.: 21011101, purchased from Changzhou SYSE Bio-Tech. Co., Ltd. (Changzhou, China). After two weeks, the model group rats were randomly divided into two groups: the NASH group and the Lingguizhugan decoction-intervened (LGZG) group (n = 6 for each group). The NASH group was fed the MCD diet, and the LGZG group was fed the MCD diet supplemented with LGZG, which was administered at 0.1 mL/10g body weight (clinical equivalent dose:16.56 g crude drug/kg) by oral gavage every day for four weeks. The Control group and NASH group rats were given an equal volume of pure sterilized water. Body weight and food intake were recorded every two days to observe the state of the rats. At the end of the experiment, the rats were fasted for 12 h, weighed, and injected with 10% chloral hydrate for anesthesia. Blood was then taken from the abdominal aorta and the serum was separated for biochemical analyses, while the liver was removed, weighed, and repacked. In addition, the ileum, colon, and feces of the ileocecal region were collected. All animal experiment procedures were approved by Shanghai University of Traditional Chinese Medicine Animal Experiment Ethics Committee (No. PZSHUTCM201218010).

### Biochemical index detection

Serum alanine aminotransferase (ALT, batch number: 01ALT210107), and aspartate aminotransferase (AST, batch number: 01AST201111) levels were analyzed and detected by an automatic biochemical analyzer (TBA-40FR, Toshiba, Tokyo, Japan). The supernatant of liver homogenate was prepared by freezing liver tissue. Then the levels of liver triglyceride (TG, batch number: 02TG201209), and liver total cholesterol (TC, batch number: 01CHOL201030) were measured using an automatic biochemical analyzer. All kits were purchased from Shanghai Huachen Biological Reagent Co., Ltd. (Shanghai, China).

### Histopathological analysis of liver

The left upper half of the liver tissues of rats were fixed in 10% neutral formalin for one week, then dehydrated, paraffin-embedded, and sectioned into 4 μm slices. Pathological changes of the liver tissues were evaluated by hematoxylin and eosin (H&E, BaSO, China) staining and the total NAFLD Activity Score (NAS score), as previously reported (Kleiner et al., 2005). The scoring system was mainly comprised of three histological features, which were evaluated semi-quantitatively: steatosis (0-3), lobular inflammation (0-3), and hepatocellular ballooning (0-2). Liver tissues used for Oil Red O (ORO) staining were frozen and embedded in optimal cutting temperature compound (OCT, SAKURA Tissue-Te, America), which were sectioned into 10 µm slices, stained with ORO and counter-stained with hematoxylin. The areas of liver ORO staining were morphologically analyzed using a StrataFAXS II image analysis system (Strata FAXS II, Vienna, Austria).

### ELISA assay

Hepatic levels of proinflammatory cytokines such as tumor necrosis factor α (TNF-α, batch number: ml002859-2), interleukin 6 (IL-6, batch number: ml102828-2), and interleukin 1β (IL-1β, batch number: ml003057-2) were measured using enzyme-linked immunosorbent assay (ELISA) kit (Shanghai Enzyme-linked Biotechnology Co., Ltd, Shanghai, China), according to the manufacturer’s protocol.

### The 16S rRNA sequencing study

The 16S rRNA of fecal samples was sequenced by Metabo-Profile Biotechnology (Shanghai) Co., Ltd. (Shanghai, China), following the manufacturer’s procedures. Briefly, total genomic DNA was extracted using the OMEGA Soil DNA Kit (M5635-02) (Omega Bio-Tek, Norcross, GA, USA). The quantity and quality of extracted DNAs were measured using a NanoDrop NC2000 spectrophotometer (Thermo Fisher Scientific, Waltham, MA, USA) and agarose gel electrophoresis, respectively. PCR amplification of the V3–V4 regions of bacterial 16S rRNA genes was then conducted. PCR amplicons were purified with Vazyme VAHTSTM DNA Clean Beads (Vazyme, Nanjing, China) and quantified using the Quant-iT PicoGreen dsDNA Assay Kit (Invitrogen, Carlsbad, CA, USA). After the individual quantification step, amplicons were pooled in equal amounts, and paired-end 2×250 bp sequencing was performed using the Illumina NovaSeq platform with the NovaSeq 6000 SP Reagent Kit (500 cycles; Illumina, San Diego, CA, USA).

The sequence data were processed using QIIME2 according to previously described methods (Gupta et al., 2022). Briefly, raw sequence data were demultiplexed, quality filtered, denoised, merged, and chimera removed. Non-singleton amplicon sequence variants (ASVs) were aligned to construct a phylogeny. Taxonomy was assigned to ASVs using the classify-sklearn naiïve Bayes taxonomy classifier against the SILVA Release 138 Database (Gupta et al., 2022). Alpha diversity indices, including the Chao1 richness estimator, Shannon diversity index, and Simpson index were calculated and visualized as box plots. Un-weighed principal coordinate analysis (PCoA) and the unweighted pair-group method with arithmetic means (UPGMA) system clustering tree for beta diversity were performed to investigate the structural variation and similarity of microbial communities across samples. The relative abundance of taxonomy at the genus level was visualized as a bar chart using R software (R Foundation for Statistical Computing, Vienna, Austria). Metastats analysis with p values less than 0.05 was used to identify significantly changed microbiota between groups. A Venn diagram was performed to obtain overlapped microbiota between pairwise compared groups (NASH vs. Control and LGZG vs. NASH), and a hierarchical cluster was used to reveal changing patterns of microbiota among groups and to identify LGZG reversed microbiota.

### Metabolomics study

Metabolomics analyses of fecal samples were performed by Metabo-Profile Biotechnology (Shanghai) Co., Ltd. (Shanghai, China), following the manufacturer’s instructions. Feces samples were thawed on an ice bath, about 5mg of each lyophilized sample was weighed and transferred to a new 1.5 mL tube. Then, 25 μL of water was added, and the sample was homogenized with zirconium oxide beads for 3 minutes, then 120 μL of methanol containing an internal standard was added to extract the metabolites. The sample was homogenized for another 3 minutes and then centrifuged at 18000 g for 20 minutes. A total of 20 μL of supernatant was transferred to a 96-well plate. The following procedures were performed using an Eppendorf epMotion Workstation (Eppendorf Inc., Hamburg, Germany). A total of 20 μL of freshly prepared derivative reagents was then added to each well. The plate was sealed and derivatization was conducted at 30°C for 60 min. After derivatization, 330 μL of an ice-cold 50% methanol solution was added to dilute the sample. Then the plate was stored at -20°C for 20 minutes, followed by 4000g centrifugation at 4°C for 30 minutes. A total of 135 μL of supernatant was then transferred to a new 96-well plate with 10 μL internal standards in each well. Serial dilutions of derivatized stock standard were added to the left wells.

Ultra-performance liquid chromatography coupled to tandem mass spectrometry (UPLC-MS/MS) system (ACQUITY UPLC-Xevo TQ-S, Waters Corp., Milford, MA, USA) was used to quantify all targeted metabolites. The following were the instrument settings. Column: ACQUITY UPLC BEH C18 1.7 µM Van Guard pre-column (2.1×5 mm) and ACQUITY UPLC BEH C18 1.7 µM analytical column (2.1×100 mm); column temperature is 40°C. The mobile phase consisted of distilled water containing 0.1% formic acid (A) and 70% acetonitrile-30% isopropanol (B). The following gradient conditions were used: 0-1.0 min, 5% B; 1.0-11.0 min, 5% B - 78% B; 11.0-13.5 min, 78% B - 95% B; 13.5-14.0 min, 95% B - 100% B; 14.0-16.0 min, 100% B; 16.0-16.1 min, 100% B - 5% B; 16.1-18.0 min, 5% B; flow rate (mL/min):0.40; and injection volume (µL):5. Additionally, the analytical quality control experiments were performed according to previously described methods (Xie et al., 2021). The pooled QC samples were prepared by mixing aliquots of the study samples such that the pooled samples broadly represented the biological average of the whole sample set. The QC samples for this project were prepared with the test samples and injected at regular intervals (after every 10-14 test samples for LC-MS) throughout the analytical run to ensure a consistently high quality of analytical results. All the QC samples, calibrators, and blank samples were analyzed across the entire sample set to diminish analytical bias.

The raw data files generated by UPLC-MS were processed using the MassLynx software (v4.1, Waters, Milford, MA, USA) to perform peak integration, calibration, and quantitation for each metabolite. Multivariate statistical analyses, including principal component analysis (PCA) and orthogonal partial least square discriminant analysis (OPLS-DA), were performed using SIMCA software (version 14.1). Besides, a 200-times permutation was conducted to validate the OPLS-DA model against over-fitting, and variable influence on projection (VIP) values of each metabolite in the OPLS-DA model was calculated. For univariate statistical analyses, a t-test or Wilcoxon test was performed to calculate p values. Finally, VIP>1 and p<0.05 were used to identify significantly changed metabolites between groups. A Venn diagram was performed to obtain overlapped metabolites between pairwise compared groups (NASH vs. Control and LGZG vs. NASH), and hierarchical clustering was used to identify the changing patterns of metabolites among groups and to identify LGZG reversed metabolites. Enrichment analyses of reversed metabolites were performed using MetaboAnalyst online tools.

### Statistical analysis

The measurement data were shown as the mean ± standard deviation (SD), and all data were statistically analyzed by one-way analysis of variance (ANOVA) followed by independent-sample-t-test using GraphPad Prism 8.4.2 (GraphPad, San Diego, CA, USA). Spearman rank correlation was performed among LGZG reversed gut microbiota, fecal metabolites, and NASH indexes. A value of p <0.05 was considered statistically significant.

## Results

### LGZG improved serum AST, ALT, and liver TG in NASH

**Figure 1:**
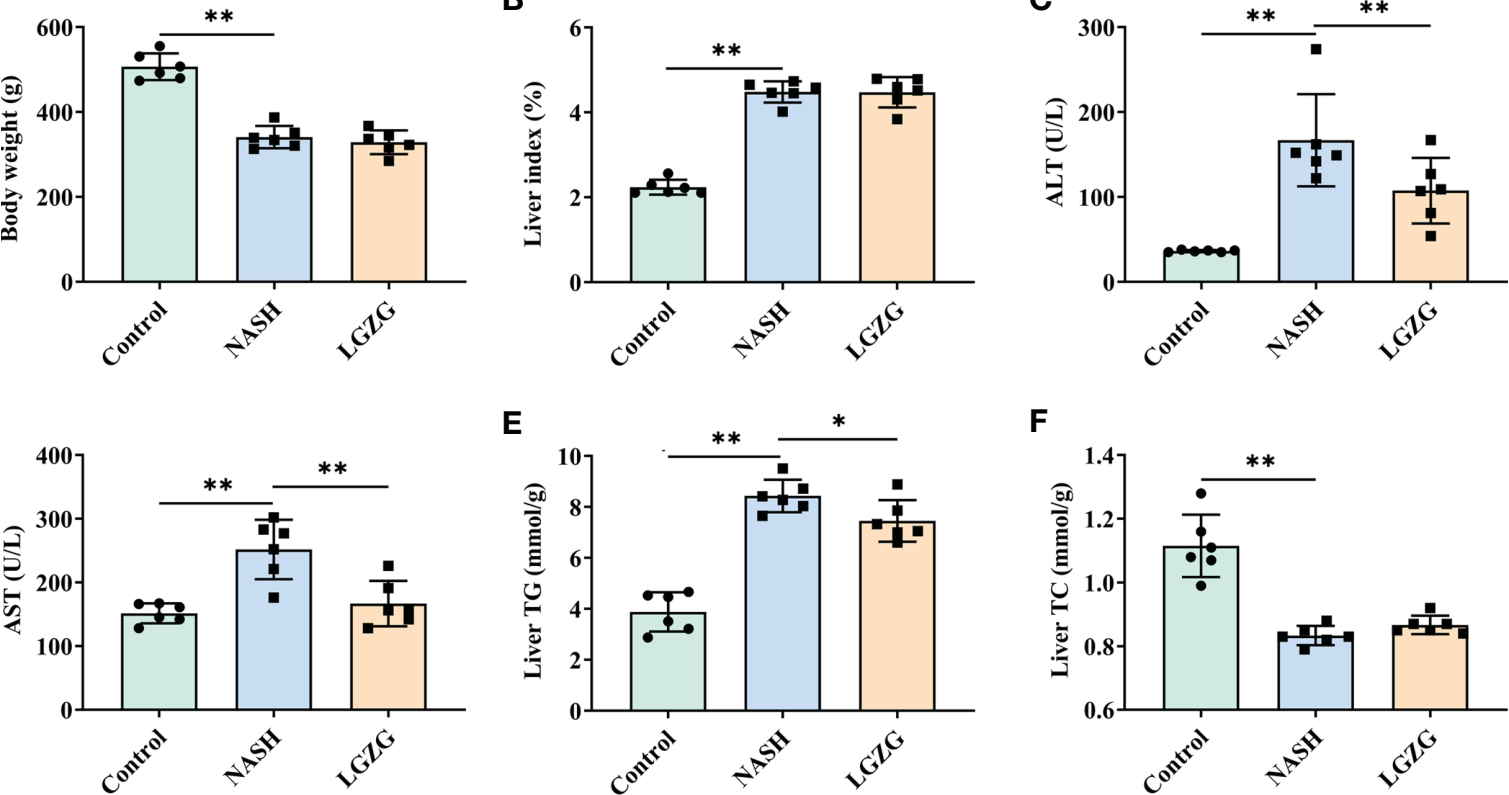
LGZG improved serum AST, ALT and liver TG in NASH. **(A)** Body weight (g), **(B)** Liver index, the ratio of liver weight to body weight (%). Livers were isolated and washed with normal saline at the end of the experiment. Livers were weighed and the liver index was calculated. **(C)** Serum AST (U/L), **(D)** Serum ALT level (U/L), **(E)**. Liver TG level (mmol/g), and **(F)**. Liver TC level (mmol/g). Serum ALT, AST and liver TG, TC levels were measured using an automatic biochemical analyzer. The green bar represents the Control group, the blue bar represents the NASH group, and the yellow bar represents the LGZG group (n = 6 per group). Data are expressed as the mean ± SD (*p < 0.05; **p < 0.01).

To evaluate the effect of LGZG on NASH, we constructed the MCD diet-induced NASH model. MCD diet-fed rats were then treated with either LGZG or vehicle for four weeks. Compared to the Control group, the NASH group showed a significant decrease in body weight and an increase in liver index (the ratio of liver weight to body weight), serum ALT and AST levels. In addition, MCD feeding significantly elevated liver TG content while decreasing liver TC content in rats. After four weeks of LGZG intervention, serum AST, ALT, and liver TG levels were significantly decreased in MCD diet-fed rats. However, body weight and liver index were not significantly changed after the decoction intervention (**Figure 1**).

### LGZG improved lipid droplets, steatosis, and inflammation in NASH

**Figure 2:**
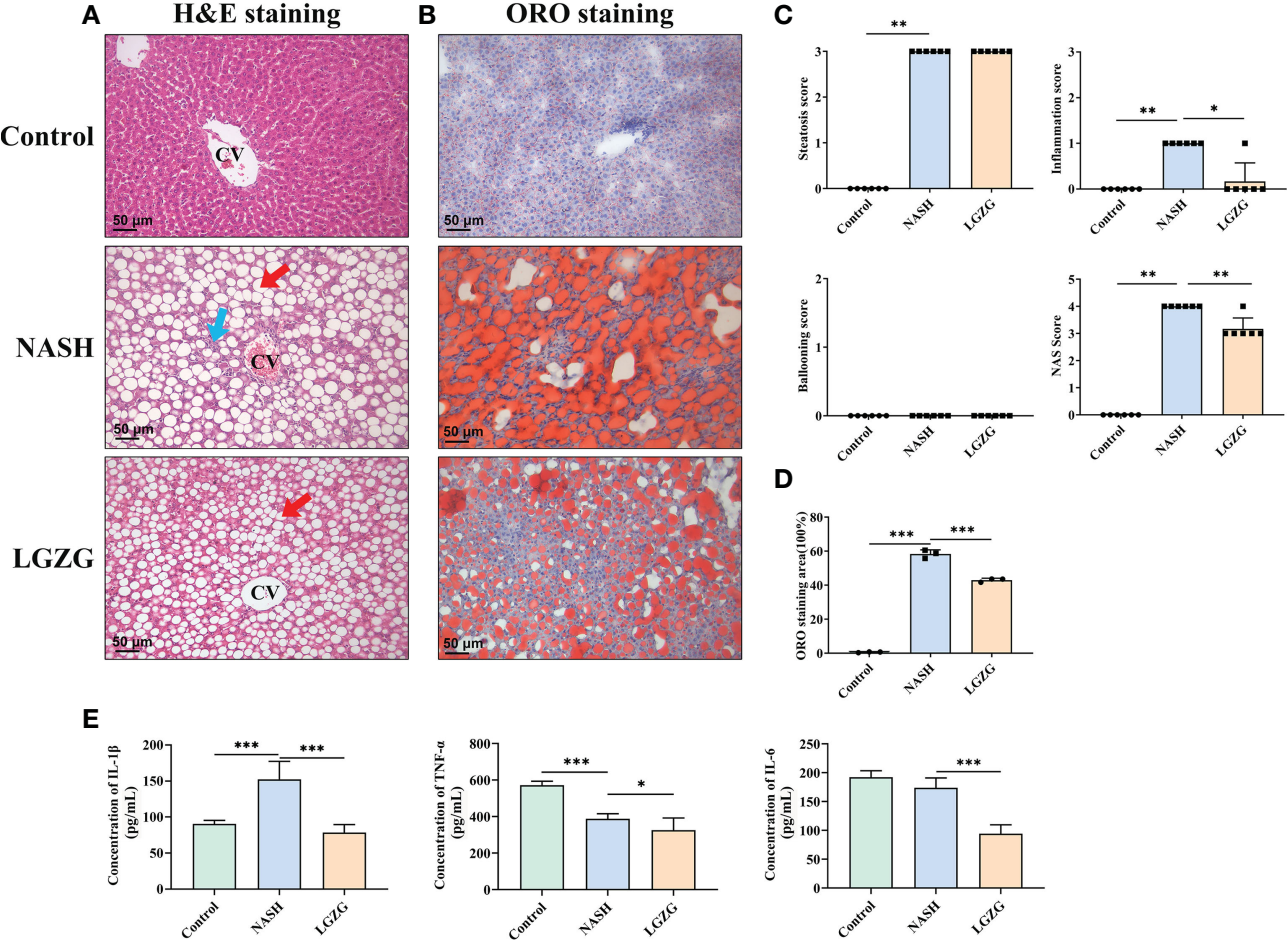
LGZG improved lipid droplets, steatosis and inflammation in NASH. **(A)** Hemoxylin & Eosin staining in liver section in the Control, NASH, and LGZG groups, respectively. The red arrow indicates macro-vesicular steatosis, and the blue arrow indicates interlobular inflammation (magnification, ×200). **(B)** Oil Red O staining in the Control, NASH, and LGZG groups, respectively (magnification, ×200). **(C)** NAFLD activity score (NAS Score) including steatosis, inflammation, and ballooning scores. **(D)** The Oil Red O staining area; data are expressed as the means ± SD (^*^p <0.05). **(E)** The expressions of pro-inflammatory cytokines IL-1β, TNFα, and IL-6 by ELISA (pg/mL). Data are presented as the mean ± SD (*p < 0.05; ** p<0.01; ***p < 0.001).

To observe the effects of LGZG on liver histological changes in NASH, a histopathological examination was performed. As shown in **Figure 2**, H&E and ORO staining revealed that steatosis, inflammation, and lipid droplets were increased in the MCD diet-induced NASH rats compared with the Control group, which were partially restored by LGZG. Moreover, the inflammation score and NAS score were significantly higher in the NASH than in the Control group, which were significantly lowered by LGZG intervention. The ORO staining areas of the LGZG group were reduced by nearly 1/3 compared with the NASH group (average 64.8% to 43.1%). Meanwhile, the level of liver proinflammatory cytokine IL-1β was significantly increased in NASH rats, while IL-1β, IL-6, and TNF-α levels were significantly decreased by the LGZG.

### LGZG partially restored the perturbation of gut microbiota in NASH

**Figure 3:**
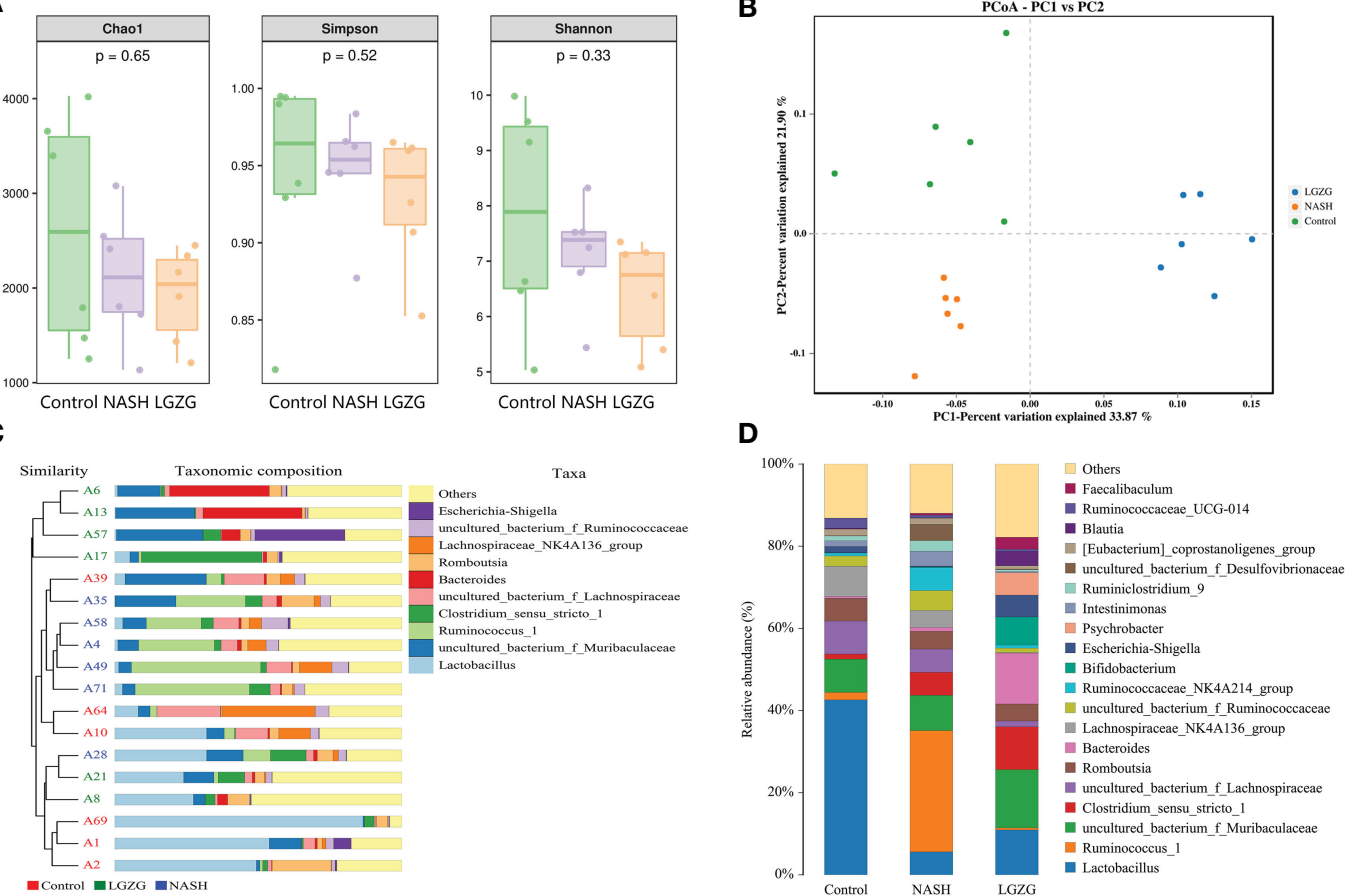
Effect of the LGZG on diversity and composition of gut microbiota. **(A)** Alpha-diversity analysis with Chao 1, Simpson, and Shannon indexes; the p values were calculated using the Kruskal-Wallis test for three group comparisons, **(B)** Principal coordinate analysis (PCoA) among the Control, NASH, LGZG groups, n=6 individuals/group, **(C)** UPGMA tree of three groups. A1, A2, A10, A39, A64, and A69 represent samples in the Control group, A4, A28, A35, A49, A58, and A71 represent samples in the NASH group, and A6, A8, A13, A17, A21, and A57 represent samples in the LGZG group. The bars in different colors represent different taxa at genus levels, **(D)** The relative abundances of gut microbiota at genus levels in the Control, NASH, and LGZG groups; the X-axis represents different groups and the Y-axis represents the relative abundance (%), while bars in different colors represent different taxa at genus levels.

The 16S rRNA sequencing was used to investigate the effect of LGZG on gut microbiota in MCD diet-induced NASH rats. The α-diversity indexes including Chao1, Shannon, and Simpson were used to determine the ecological diversity within the microbial community. As shown in **Figure 3A**, there were decreased trends of Chao1, Shannon, and Simpson in the NASH group compared with the Control group; however, there was no significant difference among the Control, NASH, and LGZG groups. To investigate structural variations of microbial communities across samples, PCoA of β-diversity was performed. The results revealed that there were distinct separations, which indicated that there might be different compositions of gut microbiota among the control, NASH, and LGZG groups (**Figure 3B**). Furthermore, UPGMA analysis revealed that the composition of gut microbiota was similar within the same group and different among groups (**Figure 3C**). To reveal the different compositions, the relative abundances of gut microbiota at the genus level were compared across samples in the three groups. As shown in **Figure 3D**, the relative abundances of many microbiotas at the genus level were different among the Control, NASH, and LGZG groups. For example, the relative abundance of *Lactobacillus* accounted for more than 40% of gut microbiota in the Control group, which decreased to about 5% in the NASH group and was restored to about 10% in the LGZG group.

**Figure 4:**
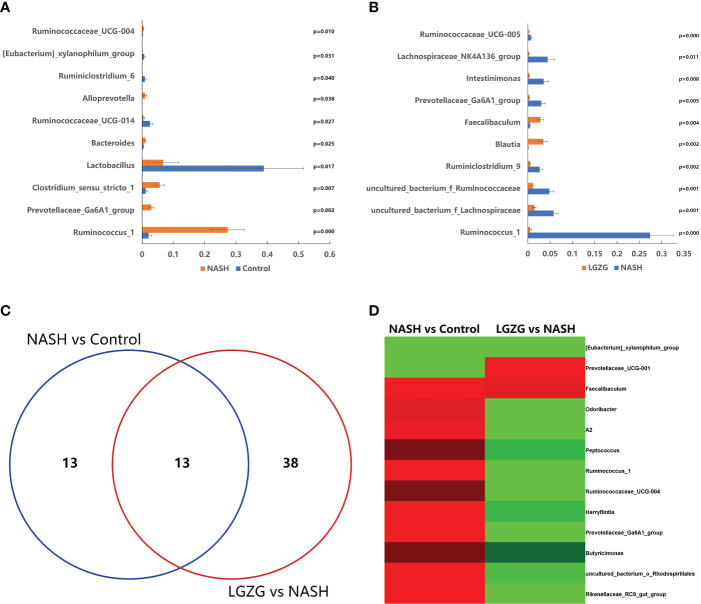
Significantly altered gut microbiota on the genus level among groups. **(A)**. The 10 representatives significantly changed genera between the Control and NASH groups; the X-axis represents relative abundance and Y-axis represents the genus name, **(B)**. The 10 representatives significantly changed genera between the NASH and LGZG groups; the X-axis represents relative abundance and the Y-axis represents the genus name, **(C)**. Venn diagram between pairwise groups (NASH vs. Control and LGZG vs. NASH), **(D)**. Hierarchical cluster of overlapped microbiotas between pairwise groups (NASH vs. Control and LGZG vs. NASH); the red color indicates up-regulated microbiota and the green color indicates down-regulated microbiota.

To identify the significantly changed microbiota at the genus level among groups, Metastats analyses with values of p < 0.05 were performed. The results revealed 26 significantly changed genera (such as *Ruminococcus_1*, *Peptococcus*, *Lactococcus*, *etc.*) between the NASH and Control groups, and 51 significantly changed genera (such as *Eisenbergiella, Intestinimonas*, *Allobaculum*, *etc.*) between the LGZG and NASH groups. The 10 representatives significantly changed genera were shown in **Figures 4A, B**, and the detailed information of all significantly changed genera was listed in **Tables 1**, **2**, and **Supplemental Table 1**. Besides, a Venn diagram was used to identify overlapped genera between the pairwise groups (NASH vs. Control and LGZG vs. NASH), with a total of 13 genera being identified (**Figure 4C**). Furthermore, hierarchical clustering was then used to identify the microbial changed patterns among groups and to identify LGZG reversed genera. As shown in **Figure 4D**, 11 genera exhibited an opposite pattern between NASH vs. Control and LGZG vs. NASH, including *Ruminococcus_1*, *A2*, *Odoribacter*, *Harryflintia*, *Peptococcus*, *Ruminococcaceae_UCG-004*, *Butyricimonas*, *Prevotellaceae_Ga6A1_ group*, *Prevotellaceae_UCG-001*, *Rikenellaceae_RC9_gut_group*, and *uncultured_ bacterium_o_Rhodospirillales*. For example, the relative abundances of *Ruminococcus _1*, *Odoribacter*, and *Butyricimonas* were increased in NASH, which was decreased after LGZG treatment. Together, the results indicated that LGZG might partially reverse the change of 11 genera in NASH.

**Table 1:** Significantly changed gut microbiota between NASH and control groups.

genus name	Log_2_(fold change)	p value
Harryflintia	4.056	0.000
Ruminococcus_1	3.711	0.000
Odoribacter	2.612	0.001
A2	2.731	0.001
Ruminococcaceae_UCG-007	-3.188	0.001
Prevotellaceae_Ga6A1_group	7.335	0.002
Faecalibaculum	6.388	0.003
uncultured_bacterium_f_Family_XIII	-1.934	0.004
Butyricimonas	1.448	0.004
Family_XIII_UCG-001	-3.431	0.007
Clostridium_sensu_stricto_1	2.284	0.007
Rikenellaceae_RC9_gut_group	5.794	0.009
Ruminococcaceae_UCG-004	1.490	0.010
Lactobacillus	-2.543	0.017
uncultured_bacterium_o_Rhodospirillales	3.414	0.018
Ruminococcus_2	-6.754	0.019
Bacteroides	1.217	0.025
Ruminococcaceae_UCG-014	-2.291	0.027
Pygmaiobacter	-2.964	0.031
[Eubacterium]_xylanophilum_group	-4.109	0.031
Lactococcus	-7.412	0.033
Alloprevotella	4.866	0.038
Prevotellaceae_UCG-001	-3.389	0.039
Ruminiclostridium_6	-7.258	0.040
uncultured_bacterium_o_Mollicutes_RF39	-8.589	0.050
Peptococcus	1.444	0.050

**Table 2:** Significantly changed gut microbiota between LGZG and NASH groups.

genus name	Log_2_(fold change)	p value
Ruminococcaceae_UCG-009	-2.594	0.000
Ruminococcaceae_UCG-010	-2.380	0.000
Ruminococcus_1	-5.777	0.000
A2	-5.032	0.000
Tyzzerella	-3.987	0.000
Odoribacter	-3.213	0.001
uncultured_bacterium_o_Clostridiales	-3.763	0.001
Ruminiclostridium	-5.492	0.001
Harryflintia	-2.362	0.001
Ruminococcaceae_UCG-004	-4.285	0.001
Anaerovorax	-3.953	0.001
uncultured_bacterium_f_Lachnospiraceae	-1.988	0.001
Allobaculum	3.786	0.001
uncultured_bacterium_f_Ruminococcaceae	-2.160	0.001
Ruminiclostridium_9	-2.413	0.002
Blautia	5.195	0.002
GCA-900066225	-1.843	0.003
uncultured_bacterium_f_Christensenellaceae	2.342	0.003
Faecalibaculum	2.687	0.004
Microcystis_PCC-7914	-2.161	0.004
Prevotellaceae_Ga6A1_group	-3.362	0.005
Oscillibacter	-2.186	0.006
uncultured_bacterium_f_Erysipelotrichaceae	1.354	0.006
Lachnospiraceae_FCS020_group	-1.626	0.006
Christensenellaceae_R-7_group	-1.459	0.006
GCA-900066575	-2.465	0.007
Eubacterium	8.232	0.007
Peptococcus	-2.313	0.008
Intestinimonas	-3.635	0.008
Aerococcus	4.182	0.008
Butyricimonas	-1.209	0.010
Lachnospiraceae_NK4A136_group	-4.472	0.011
[Eubacterium]_xylanophilum_group	-6.192	0.012
[Ruminococcus]_torques_group	7.897	0.012
Lachnospiraceae_UCG-006	-2.046	0.012
Prevotellaceae_UCG-001	4.190	0.014
Rikenellaceae_RC9_gut_group	-4.185	0.015
Adlercreutzia	1.263	0.018
Klebsiella	-1.304	0.021
Coriobacteriaceae_UCG-002	4.985	0.021
Dubosiella	2.617	0.022
Alistipes	-1.490	0.023
uncultured_bacterium_o_Rhodospirillales	-2.683	0.033
Defluviitaleaceae_UCG-011	1.126	0.035
Ruminiclostridium_5	-1.818	0.035
Ruminococcaceae_UCG-005	-2.025	0.036
UBA1819	1.956	0.036
Parvibacter	2.434	0.038
Muribaculum	2.846	0.039
Parasutterella	2.789	0.044
Eisenbergiella	3.945	0.046

### LGZG partially restored the alteration of fecal metabolites in NASH

**Figure 5:**
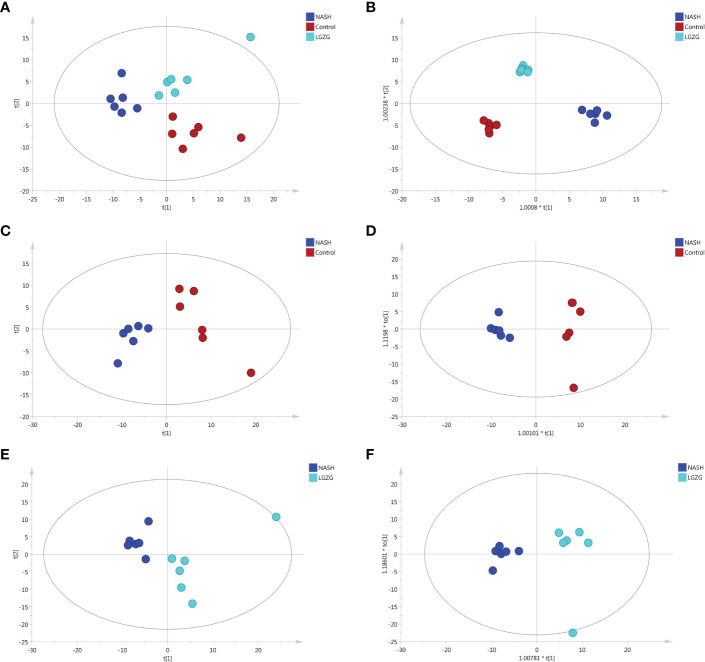
PCA and OPLS-DA plots across groups. **(A)** PCA plots among the Control, NASH and LGZG groups, **(B)** OPLS-DA plots among the Control, NASH and LGZG groups, **(C)** PCA plots between the Control and NASH groups, **(D)** OPLS-DA plots between the Control and NASH groups, **(E)** PCA plots between the LGZG and NASH groups, **(F)** OPLS-DA plots between the LGZG and NASH groups; red dots indicated samples in the Control group, blue dots indicated samples in the NASH group and green dots indicated samples in the LGZG group.

**Figure 6:**
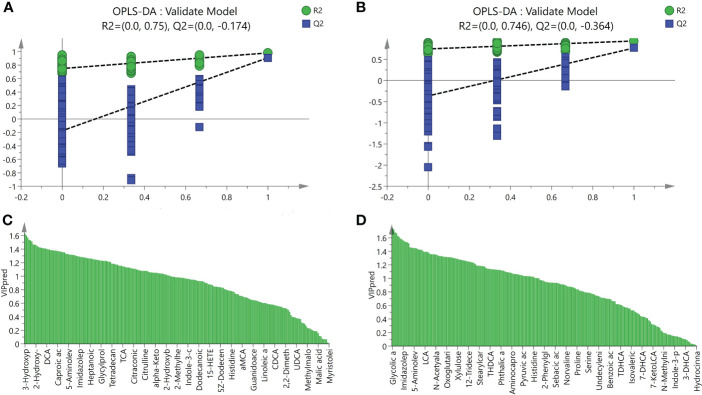
Validation model of OPLS-DA and VIP values. **(A)** The validation model of OPLS-DA for the NASH and Control groups, green dots represented R2 and blue squares represented Q2, **(B)** The validation model of OPLS-DA for LGZG and NASH groups, green dots represented R2 and blue squares represented Q2, **(C)** VIP values of OPLS-DA for the NASH and Control groups, X-axis represented names of metabolites and Y-axis represented VIP values, **(D)** VIP values of OPLS-DA for the NASH and Control groups, X-axis represented names of metabolites and Y-axis represented VIP values.

UPLC-MS was used to profile fecal metabolites across the Control, NASH, and LGZG -intervened groups. Multivariate and univariate statistical analyses were performed to identify significantly changed metabolites among groups. As shown in **Figures 5A, B**, both PCA and OPLS-DA models revealed that there were distinct separations among the Control, NASH, and LGZG groups, which indicated that profiles of metabolites exhibited different patterns among the three groups. In addition, PCA and OPLS-DA models for two pairwise groups (NASH vs. Control and LGZG vs. NASH) also revealed good separations (**Figures 5C–F**). To validate the OPLS-DA model against overfitting, two hundred times permutation tests were performed. As shown in **Figure 6A**, the results suggested good reliability of the OPLS-DA model for the Control and NASH groups with R2= (0.0,0.75), and Q2= (0.0, -0.174). Moreover, the results (**Figure 6B**) also suggested the good quality of the OPLS-DA model for the LGZG and NASH groups with R2= (0.0,0.746), and Q2= (0.0, -0.364). Furthermore, VIP values of each metabolite were obtained in OPLS-DA models (**Figures 6C, D**).

**Figure 7:**
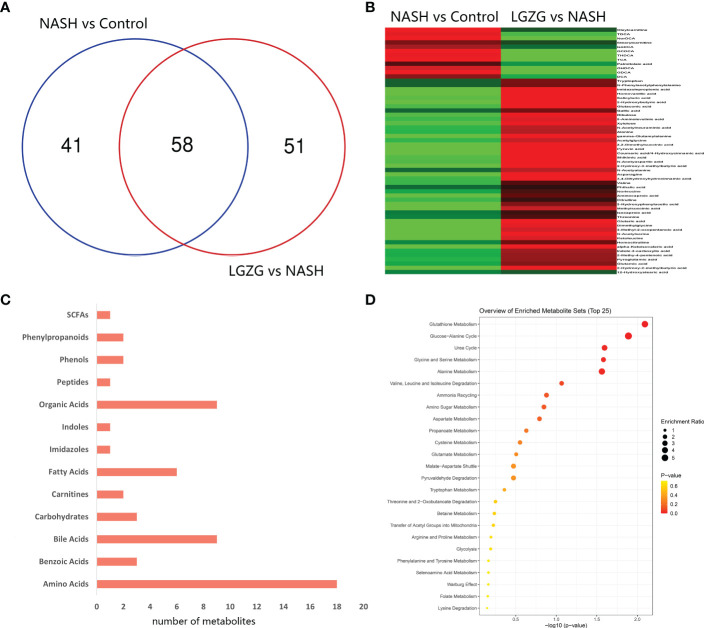
Significantly changed metabolites among groups and enrichment analysis. **(A)** Venn diagram between the two pairwise groups (NASH vs. Control and LGZG vs. NASH) overlapped 58 significant changed metabolites, **(B)** The heatmap showed the cluster analysis of 58 overlapping metabolites; the red color indicated up-regulated metabolites and the green color indicated down-regulated metabolites, **(C)** 57 LGZG-restored metabolites were grouped into 13 classes, the X-axis represented the number of changed metabolites in the same class and the Y-axis represented different classes, **(D)** Enrichment analysis of KEGG pathway for 57 LGZG-restored metabolites, the X-axis represented p value, and the Y-axis represented the name of pathway, the size of the dots represented different enrichment ratios and the color represented the different p values.

With VIP>1 and p <0.05, we obtained a total of 99 significantly changed metabolites (such as tyrosine, pyruvic acid, TDCA, *etc.*) between the NASH and Control groups, and 109 significantly changed metabolites (such as valeric acid, TCA, valine, *etc.*) between the LGZG and NASH groups. The detailed information was listed in **Supplemental Tables 2 and 3**, respectively. Using a Venn diagram (**Figure 7A**), 58 overlapped metabolites (such as DCA, TDCA, isocaproic acid, *etc.*) were obtained between pairwise groups (NASH vs. Control and LGZG vs. NASH), with the detailed information listed in **Table 3**. Moreover, hierarchical clustering revealed that 57 metabolites exhibited opposite patterns between pairwise groups (**Figure 7B**). For example, DCA and TDCA levels in NASH rats were higher than in the control group but were lowered by LGZG- intervention. Isocaproic acid was lower in the NASH group compared with the Control group, but it was increased by LGZG decoction intervention. The results indicated that LGZG decoction might reverse the alteration of metabolites in MCD diet-induced NASH. Furthermore, the 57 reversed metabolites were classified and subjected to enrichment analysis. The results revealed that most of the metabolites were amino acids, bile acids, and organic acids (**Figure 7C**, **Supplemental Table 5**), which were enriched in many Kyoto Encyclopedia and Genes and Genomes (KEGG) pathways such as glutathione metabolism, glucose-alanine cycle, glycine and serine metabolism, *etc.* (**Figure 7D**).

**Table 3:** 58 overlapped significantly changed metabolites between pairwise groups (NASH vs. Control and LGZG vs. NASH).

Metabolite	NASH vs. Control log_2_(fold change)	p value	LGZG vs. NASH log_2_(fold change)	p value
12-Hydroxystearic acid	-1.519	0.015	-1.082	0.041
2,2-Dimethylsuccinic acid	-3.996	0.029	4.104	0.073
2-Hydroxy-2-methylbutyric acid	-3.917	0.005	3.044	0.005
2-Hydroxy-3-methylbutyric acid	-4.393	0.002	6.487	0.002
2-Hydroxybutyric acid	-4.700	0.004	5.563	0.002
2-Methy-4-pentenoic acid	-1.979	0.000	1.751	0.002
3,4-Dihydroxyhydrocinnamic acid	-2.367	0.001	4.309	0.065
3-Hydroxyphenylacetic acid	-4.606	0.002	1.516	0.002
3-Methyl-2-oxopentanoic acid	-3.308	0.002	2.423	0.009
5-Aminolevulinic acid	-2.351	0.002	2.537	0.002
Acetylglycine	-2.246	0.002	2.280	0.002
Alanine	-2.142	0.002	2.087	0.000
alpha-Ketoisovaleric acid	-3.498	0.002	2.951	0.004
Aminocaproic acid	-2.941	0.002	1.229	0.015
Asparagine	-5.895	0.017	10.477	0.004
Citrulline	-2.351	0.002	0.769	0.065
Coumaric acid/4-Hydroxycinnamic acid	-3.891	0.002	5.455	0.002
DCA	1.625	0.004	-1.984	0.003
Dimethylglycine	-3.647	0.002	2.679	0.009
Gallic acid	-1.208	0.005	1.414	0.026
gamma-Glutamylalanine	-8.268	0.004	8.356	0.004
GCDCA	6.696	0.013	-4.716	0.026
GDCA	5.115	0.005	-7.027	0.013
GHDCA	2.438	0.002	-3.320	0.009
Glutaconic acid	-2.575	0.002	3.025	0.004
Glutamic acid	-1.940	0.002	1.579	0.009
Glutaric acid	-4.877	0.002	3.276	0.002
Homocitrulline	-1.585	0.002	1.332	0.002
Homovanillic acid	-4.701	0.005	6.089	0.019
Imidazolepropionic acid	-3.935	0.004	4.875	0.002
Indole-3-carboxylic acid	-2.009	0.041	1.845	0.015
Isocaproic acid	-1.436	0.002	0.823	0.010
isoDCA	1.932	0.009	-1.281	0.026
Ketoleucine	-3.001	0.002	2.527	0.015
Methylsuccinic acid	-3.758	0.002	2.282	0.002
N-Acetyalanine	-1.236	0.004	2.163	0.004
N-Acetyaspartic acid	-2.641	0.009	3.978	0.002
N-Acetylneuraminic acid	-2.221	0.004	2.152	0.002
N-Acetylserine	-4.742	0.002	4.083	0.002
NorDCA	3.286	0.002	-3.310	0.002
Norleucine	-1.928	0.002	0.912	0.065
N-Phenylacetylphenylalanine	-1.141	0.009	1.408	0.007
Oleylcarnitine	3.209	0.004	-0.990	0.091
Palmitoleic acid	1.037	0.044	-1.731	0.004
Phthalic acid	-1.243	0.002	0.605	0.065
Pyroglutamic acid	-2.116	0.002	1.731	0.002
Pyruvic acid	-3.672	0.002	5.290	0.002
Ribulose	-2.650	0.004	2.780	0.004
Salicyluric acid	-2.801	0.000	3.317	0.002
Shikimic acid	-2.703	0.015	4.066	0.002
Stearylcarnitine	1.483	0.009	-0.892	0.091
TCA	4.139	0.002	-6.419	0.029
TDCA	2.666	0.002	-2.581	0.004
THDCA	2.709	0.009	-3.981	0.041
Threonine	-1.526	0.002	1.004	0.002
Tryptophan	-1.138	0.009	1.429	0.009
Valine	-2.325	0.002	1.059	0.004
Xylulose	-2.777	0.002	2.754	0.004

### Gut microbiota and metabolites restored by LGZG were correlated with NASH indexes

**Figure 8:**
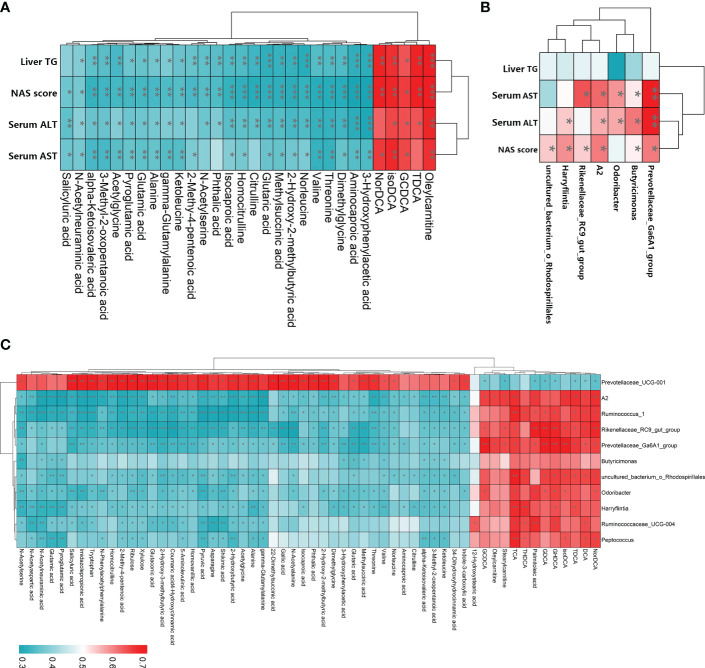
Spearman’s correlation analysis among LGZG reversed gut microbiota, fecal metabolites, and NASH indexes. **(A)** Correlation between LGZG reversed fecal metabolites and NASH indexes, the X-axis represented metabolites, and the Y-axis represented NASH indexes, **(B)** Correlation between LGZG reversed gut microbiota and NASH indexes, the X-axis represented gut microbiota, and the Y-axis represented NASH indexes, **(C)** Correlation between LGZG reversed metabolites and gut microbiota, the X-axis represented metabolites, and the Y-axis represented gut microbiota, the red color showed the positive correlation, and the green color showed the negative correlation, *p < 0.05, **p < 0.01, ***p < 0.001.

To examine the relationships among LGZG-restored gut microbiota, fecal metabolites, and NASH indexes, Spearman’s correlation analyses were performed. The results revealed that most of the LGZG restored gut microbiota and its metabolites were significantly correlated with NASH indexes (**Figures 8A, B**). For example, the fecal metabolites oleylcarnitine and isoDCA were positively correlated with liver TG, NAS score, serum AST and ALT, and TDCA was positively correlated with liver TG, NAS score, and serum ALT; whereas, valine and alanine were negatively correlated with liver TG, NAS score, serum AST and ALT. In addition, the gut microbiota *Prevotellaceae_ Ga6A1_group*, *A2*, and *Butyricimonas* were positively correlated with the NAS score, serum AST and ALT. Interestingly, we also observed that most of the LGZG-restored gut microbiota were significantly correlated with LGZG -restored fecal metabolites (**Figure 8C**). For example, *Ruminococcus_1*, *Odoribacter*, and *Peptococcus* were positively correlated with some bile acids such as DCA, TDCA, TCA, *etc.*, but negatively correlated with other fecal metabolites such as alanine, asparagine, glutamic acid, *etc.* The results indicated that LGZG might improve NASH partially by modulating gut microbiota and correlated metabolites.

## Discussion

**Figure 9:**
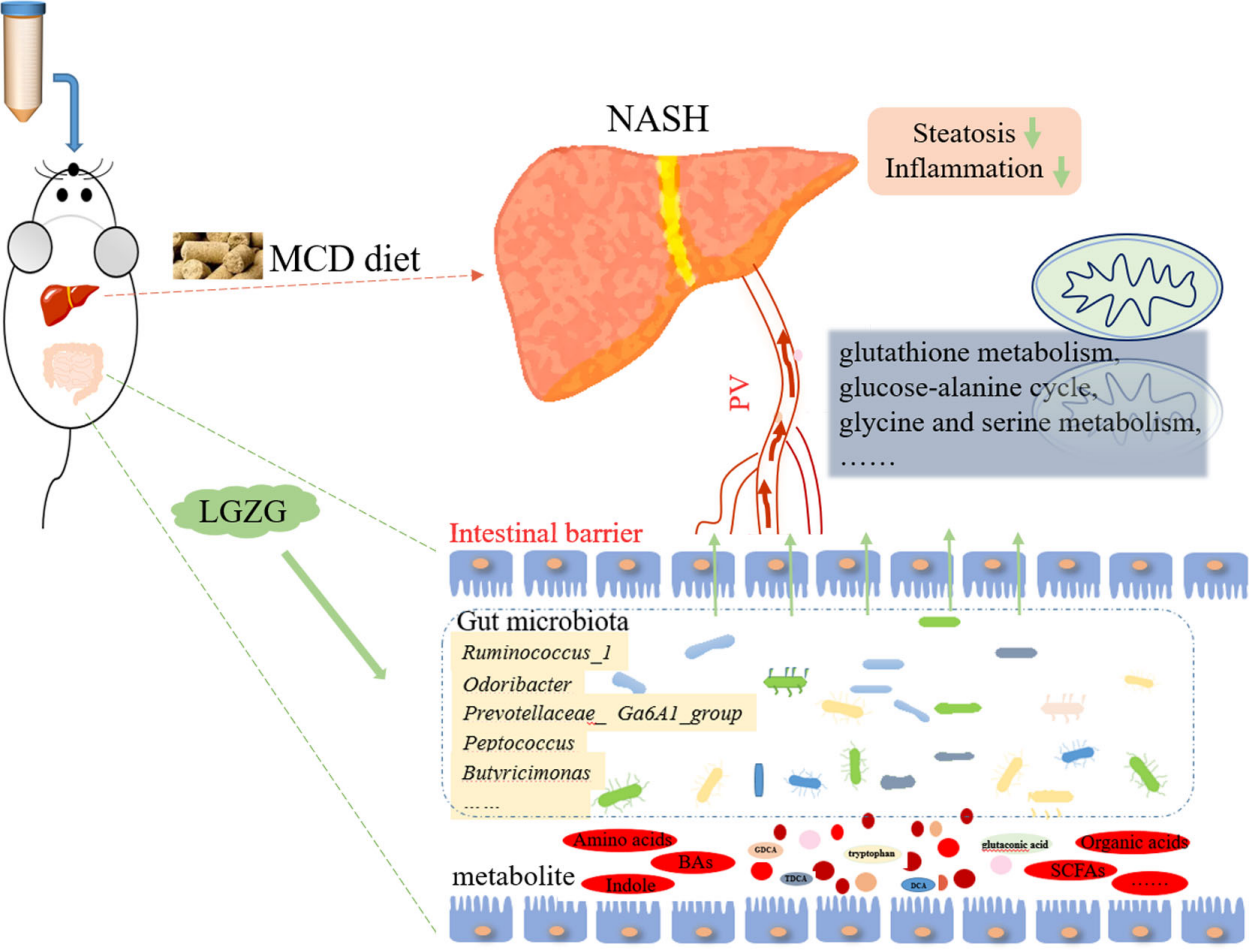
Potential mechanisms of LGZG against NASH.

The effect of LGZG on NASH and its underlying mechanisms remained unclear. In the present study, MCD diet-induced NASH and LGZG-intervened models were constructed to evaluate the effects of LGZG on NASH. Gut microbiota and metabolites in fecal samples were profiled to uncover the mechanisms from the perspective of gut microbiota. Our results suggested that LGZG might improve NASH partially by modulating gut microbiota and correlated metabolites **Figure 9**.

Accumulating evidence has demonstrated that gut microbiota perturbation plays an important role in the progression of NAFLD/NASH, so the restoration of the gut microbiota may be of therapeutic benefit for some of the diseases (Chen and Vitetta, 2020). TCMs have multi-targets, multi-channels, and multi-component synergistic actions, which have been widely used for the treatment of NAFLD/NASH for a long time (Liu et al., 2017). Emerging evidence has reported that gut microbiota might be therapeutic targets of TCMs in treating NAFLD/NASH (Han et al., 2021; Hu et al., 2021). In the present study, we identified 11 gut genera that might be therapeutic targets of LGZG in treating NASH, including *Ruminococcus_1*, *A2*, *Odoribacter*, *Harryflintia*, *Peptococcus*, *Ruminococcaceae _UCG-004*, *Butyricimonas*, *Prevotellaceae_Ga6A1_group*, *Prevotellaceae_UCG-001*, *Rikenellaceae_RC9_gut_group*, and *uncultured_ bacterium_o_Rhodospirillales.* It is noted that several identified genera have been implicated in NAFLD/NASH. *Odoribacter* has been reported to be correlated with fibrosis in choline-deficient, high-fat-diet-induced NASH mice (Yamamoto et al., 2021), and showed a significantly higher abundance in high-fat-diet-induced NAFLD mice (Hu et al., 2022). In the present study, we observed that the relative abundance of *Odoribacter* was increased in MCD diet-induced NASH, which was decreased by LGZG intervention. In addition, accumulating evidence suggested that *Ruminococcaceae* might play a key role in the progression of NAFLD/NASH. Lee et al. reported that *Ruminococcaceae* and *Veillonellaceae* might be the main microbiota associated with fibrosis severity in non-obese NAFLD patients, and *Ruminococcaceae* was gradually depleted with worsening fibrosis severity (Lee et al., 2020). However, other researchers found that the abundance of *Ruminococcus* was significantly elevated in NASH patients with severe fibrosis (Boursier et al., 2016). These inconsistent results might be due to different subjects observed in the studies. An animal study revealed that the relative abundance of *Ruminococcaceae* was elevated in high fat diet-induced NAFLD rats, and a TCM formula called Shugan Xiaozhi decoction exerted beneficial effects in treating NAFLD with decreased abundance of *Ruminococcaceae* and other gut microbiota (Yang et al., 2022). Interestingly, we also noticed similar results in the present study. The relative abundances of *Ruminococcaceae_UCG-004* and *Ruminococcus_1* were elevated in MCD diet-induced NASH, which was decreased by LGZG intervention. These results indicated that the gut microbiota reversed by LGZG might play an important role in treating NASH. Further investigation of specific genera may identify novel therapeutic targets for NASH.

Besides the changes in gut microbiota, many metabolites produced by different gut microbiota have emerged as important factors in modulating the pathological process of metabolic diseases (Ji et al., 2019; Duttaroy, 2021; Jia et al., 2021). In the present study, we identified several fecal metabolites including bile acids (BAs), amino acids (AAs), short-chain fatty acids (SCFAs), *etc.* It is known that BAs may regulate multiple physiological and pathological processes, implicating a role in the progression of NAFLD/NASH (Arab et al., 2017; Ji et al., 2019; Fiorucci et al., 2020). In the intestine, primary BAs are deconjugated and dehydroxylated by gut microbiota to main secondary BAs, including deoxycholic acid (DCA) and lithocholic acid (LCA) (Chow et al., 2017). A previous study reported that sucralose consumption increased the abundance of *Bacteroides* and *Clostridium*, which produced DCA accumulation in the feces, serum, and liver, and hepatic DCA led to sucralose-induced NAFLD in mice (Shi et al., 2021). Jiao, et al. reported that farnesoid X receptor antagonist DCA was increased, while agonist CDCA was decreased in NAFLD (Jiao et al., 2018). Another study reported that patients with NASH and clinically significant fibrosis had higher serum DCA levels when compared with healthy volunteers (Smirnova et al., 2022). In addition, taurodeoxycholate (TDCA) and glycodeoxycholate (GDCA) which belong to conjugated 12α-hydroxylated (12α-OH) BAs, have been reported to be significantly elevated in NAFLD/NASH patients. A clinical study reported that GDCA were significantly positively associated with HOMA-IR individually (Ginos et al., 2018), and TDCA inhibits various inflammatory responses suggesting potential clinical application (Choi et al., 2021). TDCA and GDCA administration promoted liver fibrosis in mice (Xie et al., 2021). The findings of the previous studies were partly consistent with our results. Here, we reported that fecal BAs, including DCA, TDCA, and GDCA, were significantly elevated in MCD diet-induced NASH rats, which were decreased by LGZG. However, the in-depth mechanisms of identified metabolites in treating NASH need further investigation, which may find novel therapeutic approaches to NASH.

Accumulating studies have demonstrated that amino acids might play a role in the process of NAFLD/NASH, but the relationship hasn’t been elucidated. It is reported that dysregulation of amino acids and choline contributed to lipid accumulation and chronic inflammation in NAFLD (Chen and Vitetta, 2020). A study revealed that dietary essential amino acids ameliorated hepatic steatosis by inducing polyubiquitination of Plin2 in NAFLD mice (Zhang et al., 2022). Another study reported that plasma concentration of several amino acids (including branched-chain and aromatic amino acids) was altered in NAFLD, and the alteration of specific amino acids (such as alanine, glutamate and glycine) might be used for early detection of NAFLD (Trico et al., 2021). Meanwhile, gut microbiota modulated amino acid metabolism have been implicated in NAFLD/NASH. Latest study revealed that targeting keystone species (*P. loveana*, *A. indistinctus*, and *D. pneumosintes*) helps restore the dysbiosis of butyrate-producing bacteria, lead to enhance the AAs synthesis and reduce AAs consumptions in NAFLD (Wu et al., 2022). In the present study, we noted a batch of amino acids (including glutamic acid, tryptophan, valine, *etc.*) and their correlated gut microbiota were altered in NASH and restored by LGZG intervention. Besides, KEGG pathway analysis showed that 57 significantly altered metabolites were mainly enriched in amino acid metabolic pathways, such as glutathione metabolism, glycine and threonine metabolism, and tryptophan metabolism. Glutathione (GSH) is a tripeptide thiol antioxidant composed of the amino acid glutamic acid, cysteine, and glycine, which can modulate cell proliferation, apoptosis, immune function, and treat liver diseases (Mari et al., 2020). A clinical study recently examined the therapeutic effects of oral administration of GSH in patients with NAFLD. After 4 months of treatment with GSH, patients showed a significant decrease in ALT levels, as well as a decrease in triglyceride, non-esterified fatty acid and ferritin levels, suggesting a potential therapeutic effect of oral GSH in patients with NAFLD (Honda et al., 2017). Impaired glycine biosynthesis was observed in NASH patients and mouse models resulting in increased hyperlipidemia and steatohepatitis, along with mitochondrial dysfunction and inhibition of fatty acid β-oxidation (FAO). However, glycine-based compounds improved FAO pathway, stimulated the new GSH synthesis, improved the gut microbiota diversity and steatohepatitis *via* targeting NF-κB and TGFβ (Alves et al., 2019; Rom et al., 2020). Glutamic acid is also involved in the synthesis of glutathione (GSH), which can combat oxidative stress, and reduce the release of inflammatory cytokines in diet-induced NAFLD animals (Lin et al., 2014). Consistently, we observed that glutamic acid was lowered in NASH and restored by LGZG decoction, and its negatively correlated gut microbiota (such as *Butyricimonas* and *Odoribacter*) was increased in NASH and decreased by LGZG. Besides, valine was reported to improve liver diacylglycerols, and reduce NASH histology with profound hepatoprotective effects on oxidative stress and inflammatory proteins (Gart et al., 2022). However, another study reported that high levels of valine could decrease gut microbiota (such as *Fusobacteriota* and *Deferribacterota)* abundances and revealed the adverse metabolic response to NAFLD, which suggested reducing dietary valine as a new approach to preventing NAFLD of laying hens (Jian et al., 2021). Here, we reported that amino acid valine was lowered in NASH and restored by LGZG. We also noticed that gut microbiota *Prevotellaceae_UCG-001* which was positively correlated with valine, was decreased in NASH and increased by LGZG. Additionally, another amino acid tryptophan was reported to ameliorate fructose-fed NAFLD mice (Ritze et al., 2014). Tryptophan could be metabolized to indole by tryptophanase, which is produced by gut microbiota such as *Prevotella*, *Bacteroides* and *Escherichia* (Jin et al., 2014). Furthermore, indole and indole derivatives have been shown to have anti-inflammatory effects (Beaumont et al., 2018). Alterations of metabolites in the tryptophan pathway have been implicated in various inflammation related diseases such as cancer, inflammatory bowel diseases, and cardiovascular diseases (Sofia et al., 2018). A study reported that indole supplementation ameliorated MCD-induced NASH in mice (Zhu et al., 2022). These results indicated that tryptophan and its metabolites might exert a protective role in NAFLD/NASH. Our results were partly consistent with these findings. In the present study, we observed that tryptophan was decreased in NASH rats and restored by LGZG, and several correlated microbiotas such as *Prevotellaceae_UCG-001* were altered. The role of these metabolites and its correlated gut microbiota in NASH is worthy of further investigation. In all, our findings suggested that LGZG treatment affected gut microbiota-mediated amino acid metabolism might be the potential targets on NASH, which needed to be further elucidated in future studies.

In addition, SCFAs are the most plentiful bacterial metabolites derived from intestinal bacteria, which have been implicated in NAFLD/NASH (Bashiardes et al., 2016). SCFAs-mediated activation of GRP43 signaling in adipose tissue promotes energy expenditure and inhibits fat accumulation in adipose tissue and even in the liver (Kimura et al., 2013). Most SCFAs are utilized in the gut, but some amount is transported to the bloodstream *via* the portal vein and be channeled into the tricarboxylic acid (TCA) cycle and become source of energy (Kolodziejczyk et al., 2019). One of the mechanisms of action of SCFAs to limit NASH is by reducing inflammatory signals (Maslowski et al., 2009). It is reported that SCFAs reduced MCD-induced hepatic aggregation of macrophages and proinflammatory responses in NASH mice (Deng et al., 2020). A human study reported that NASH patients were characterized by a different gut microbiome composition with higher fecal SCFA levels and higher abundance of SCFA-producing bacteria (Rau et al., 2018). Here, we noticed that isocaproic acid and its positively correlated gut microbiota *Prevotellaceae_UCG-001* was decreased in NASH and restored by LGZG. Isocaproic acid was reported to be produced by *Clostridia* and *Peptostreptococcus anaerobius* from Leucine metabolism and participated in the energy metabolism (Britz and Wilkinson, 1982). Therefore, the correlation between isocaproic acid and NASH will be worth exploring in the future.

Moreover, we performed Spearman rank correlation analysis to identify relationships among gut microbiota, fecal metabolites, and NASH indexes. Notably, we observed that many metabolites reserved by LGZG were significantly correlated with gut microbiota and NASH indexes. Besides, gut microbiota restored by LGZG were significantly correlated with NASH indexes. For example, changes of *Prevotellaceae_Ga6A1_ group* and *Butyricimonas* were positively correlated with the NAS score, serum AST and ALT. Meanwhile, we found that GDCA was positively correlated with five genera (such as *Prevotellaceae_Ga6A1_group* and *Odoribacter*), and TDCA was positively correlated with six genera (such as *Ruminococcus_1* and *Prevotellaceae_Ga6A1_group)* and three NASH indexes (including liver TG, serum ALT, and NAS score). Together, the results indicated that LGZG might restore the dysbiosis of gut microbiota and influence related metabolites to treat NASH.

Overall, using gut microbiome and fecal metabolomics, we identified several gut microbiota and abundant fecal metabolites reversed by LGZG in treating NASH. Our results suggested that LGZG might improve NASH by modulating gut microbiota and their correlated metabolites, which broadened our knowledge of the mechanisms of LGZG in treating NASH. However, further investigation needs to be performed, which may identify novel therapeutic targets of NASH.

## Data availability statement

The original contributions presented in the study are publicly available. This data can be found here: https://www.ncbi.nlm.nih.gov/sra/PRJNA894781, PRJNA894781.

## Ethics statement

The animal study was reviewed and approved by Shanghai University of Traditional Chinese Medicine Animal Experiment Ethics Committee.

## Author contributions

WZ, FL, and GJ contributed to conception, and designed the entire research project. XW, KW, and ZZ contributed to the animal experiments and pharmacology research. MZ, FL, and YD completed data analysis of the gut microbiota and metabolism. MZ and XW drafted the manuscript. WZ and FL reviewed the results, made revisions and contributed equally as corresponding authors. All authors contributed to the article and approved the submitted version.
