# Supplementary figures and images for: Lingguizhugan decoction improves non-alcoholic steatohepatitis partially by modulating gut microbiota and correlated metabolites
Source: Front Cell Infect Microbiol. 2023 Jan 26;13:1066053. doi: 10.3389/fcimb.2023.1066053 (PMC9908757; doi:10.3389/fcimb.2023.1066053)

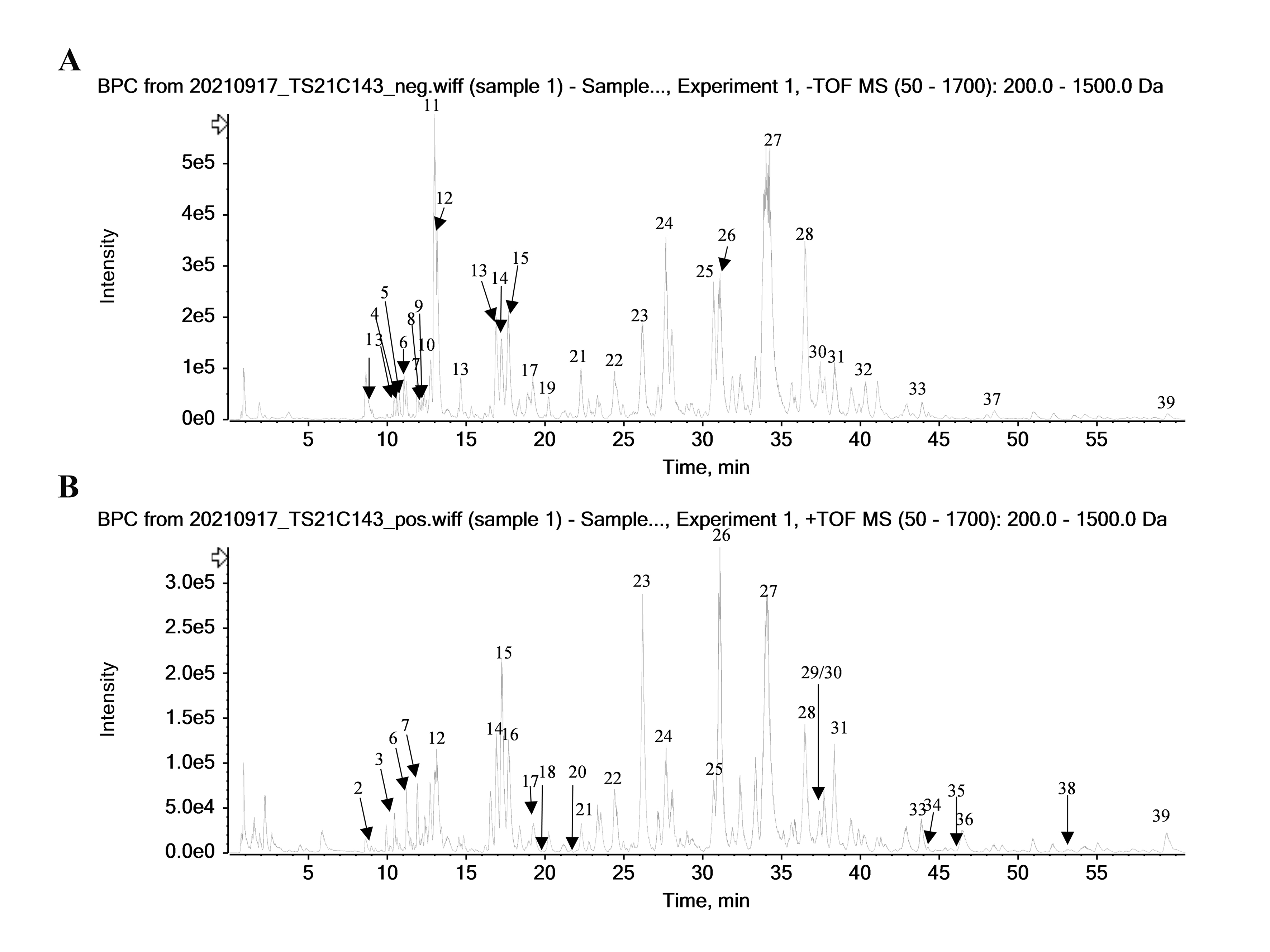

Supplement: Supplementary file 2 [file DataSheet_2.zip › Supplemental Figure 1.JPEG]
